# MRI in deciphering the superficial spread of cervical squamous cell carcinoma into the uterine corpus: A case report and literature review

**DOI:** 10.18632/oncoscience.668

**Published:** 2026-07-29

**Authors:** Naina Kumar, Aparna Jarathi, Shailaja Prabhala, Annapurna Srirambhatla

**Affiliations:** ^1^Department of Obstetrics and Gynecology, All India Institute of Medical Sciences, Bibinagar, Hyderabad, Telangana 508126, India; ^2^Department of Radiodiagnosis, All India Institute of Medical Sciences, Bibinagar, Hyderabad, Telangana 508126, India; ^3^Department of Pathology and Laboratory Medicine, All India Institute of Medical Sciences, Bibinagar, Hyderabad, Telangana 508126, India

**Keywords:** postmenopausal bleeding, MRI, p16, p63, ER, PR

## Abstract

We report a deceptive presentation of Squamous Cell Carcinoma of the Cervix in a 54-year-old postmenopausal woman who presented with postmenopausal bleeding associated with lower abdominal pain for a 3-month duration, with a normal-looking ectocervix. She was diagnosed with squamous cell carcinoma on a pap smear and cervical biopsy. Magnetic resonance imaging revealed an irregular endometrial cavity contour, and a T2 hyperintense lesion was seen (2.3 × 0.8 cm) involving the posterior lip of the cervix at the junction of stroma and endocervix with a mural spread along the uterine myometrium. Histopathology after Wertheim’s hysterectomy revealed a squamous cell carcinoma of the endocervix, Stage pT1b3, that had spread superficially to the endometrium, myometrium, and left salpinx. The detection of immunohistochemical expression of p16, p63, and negative for ER and PR in the lesions of the cervix and the endometrium suggests that these two lesions are etiologically related, favoring the possibility of endometrial SCC secondary to cervical SCC.

## INTRODUCTION

Cervical carcinoma is the second most common malignancy affecting females in India [1]. Cervical Squamous Cell Carcinoma (SCC) direct superficial spread into the uterine endometrium is an unusual and poorly understood phenomenon. The Federation of Gynecology and Obstetrics (FIGO) 2018 classification has not stated or included the involvement of myometrium and adnexa in any of the stages [2]. Therefore, the superficial spread of SCC into the endomyometrium presents a diagnostic challenge and warrants further investigation. In this case report, we present a rare case of superficially spreading squamous cell carcinoma of the cervix into the uterine endomyometrium in a 54-year-old postmenopausal woman.

## CASE REPORT

A 54-year-old woman presented to the gynecology outpatient department with postmenopausal bleeding and lower abdominal pain persisting for the past three months. She was para 3, living 3 (all spontaneous vaginal deliveries), and had attained menopause 15 years ago. She was a known hypertensive for the past 10 years, on regular follow-up with a general physician, and was maintained on a fixed-dose combination of Telmisartan 40 mg and Amlodipine 5 mg. She had no prior surgical history, no family history of malignancies, and no other significant comorbidities. Her general condition was fair, and her vitals were normal, with an unremarkable abdominal examination. Pelvic examination showed a hypertrophied ectocervix with a granular appearance, the uterus was 8 weeks in size, and a 3 × 2 cm fibroid was felt arising from the posterior wall of the uterus. The routine blood tests and serology were normal. Her pap smear and subsequent cervical biopsy both revealed squamous cell cancer. The clinical staging of our patient was FIGO 2018 stage 1A. Further evaluation by magnetic resonance imaging (MRI) showed an irregular endometrial cavity contour; a T2 hyperintense lesion was seen (2.3 × 0.8 cm) involving the posterior lip of the cervix at the junction of stroma and endocervix with a mural spread along the uterine myometrium (Figure 1A–1C: MRI images: 1A: Double Asterisk: T2 hyperintense mass involving the uterus’s anterior and posterior myometrium. Yellow Asterisk: Uterine fibroid 1B: Diffusion weighted 1C: ADC mapping showing restricted diffusion in the mass involving the lower uterine body). Diffuse restriction was noted in the endocervical lesion. Because of the MRI findings, the patient’s provisional diagnosis of superficially spreading squamous cell carcinoma of the cervix into the uterine endomyometrium was upgraded with FIGO 2018 upstaged to stage 1B3.

**Figure 1:**
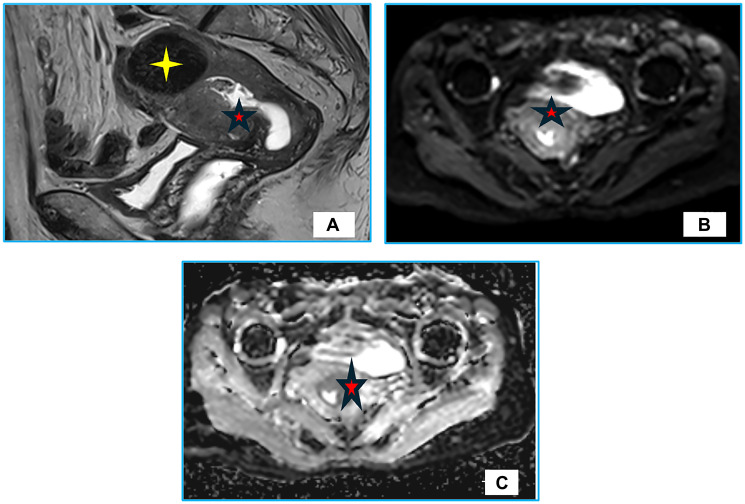
MRI images. (**A**) Double Asterisk: T2 hyperintense mass involving both the anterior and posterior myometrium of the uterine body. Yellow Asterisk: uterine fibroid. (**B**) Diffusion weighted. (**C**) ADC map: restricted diffusion in the mass involving the lower uterine body.

The patient underwent a radical hysterectomy (Figure 2: Post-surgical gross specimen of uterus with bilateral adnexa). (Figure 3: Post-surgical specimen cut section showing the entire endometrial cavity and endocervical with infiltrative tumor growth. A calcified fundal fibroid measuring 4 × 4 × 3 cm is present). Her postoperative recovery was good. Histopathology revealed stage pT1b3 squamous cell carcinoma of the cervix with a superficial spread to the endomyometrium. The myometrial invasion was less than 50% and a nodule of 0.5 cm at the left fimbrial end showed SCC (Figure 4: Nodule in the left fallopian tube at the fimbrial end with tiny tumor deposits (blue arrows) (H and E stain, 400X). Immunohistochemistry showed the tumor cells were positive for p16 and p63, and negative for estrogen receptor (ER) and progesterone receptor (PR) (Figure 5A, 5B: 5A shows p16 positivity, 5B shows p63 positivity; Figure 5C, 5D: 5C shows ER negativity, 5D shows PR negativity). The final decision to withhold further adjuvant therapy was made following a multidisciplinary team discussion at the state oncology board. Consequently, the patient did not receive chemoradiation. She is under regular follow-up and is clinically well, with no evidence of disease recurrence eighteen months post-surgery.

**Figure 2:**
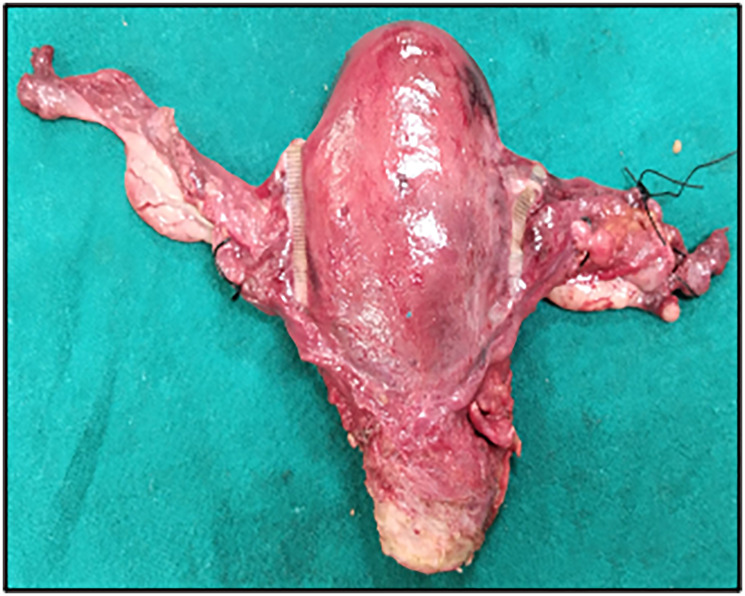
Post-surgical gross specimen of the uterus with bilateral adnexa.

**Figure 3:**
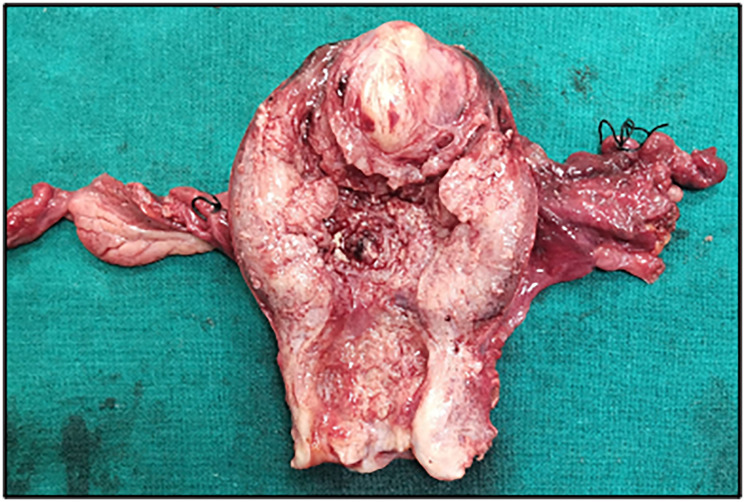
Post-surgical specimen cut section showing the entire endometrial cavity and endocervix with infiltrative tumor growth. A calcified fundal fibroid measuring 4 × 4 × 3 cm is present.

**Figure 4:**
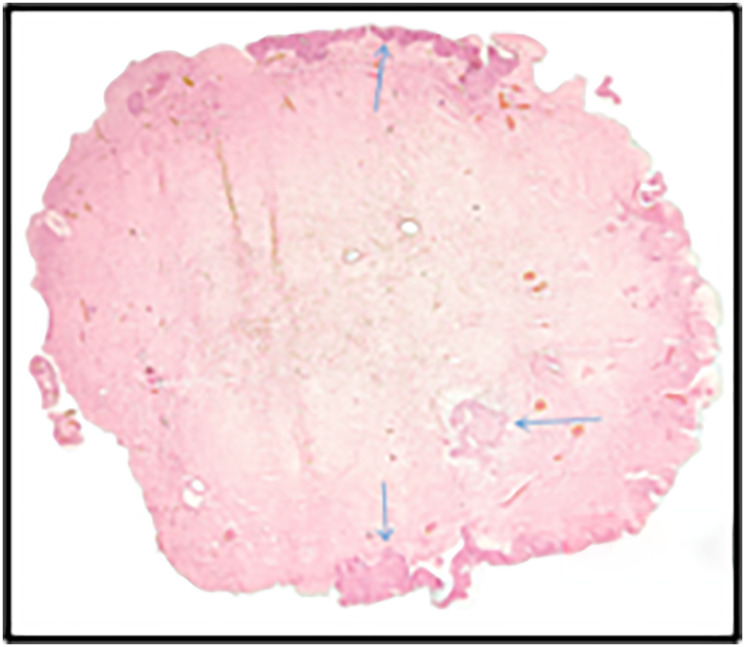
Nodule in the left fallopian tube at the fimbrial end with tiny tumor deposits (blue arrows). (H and E stain, 400X).

**Figure 5:**
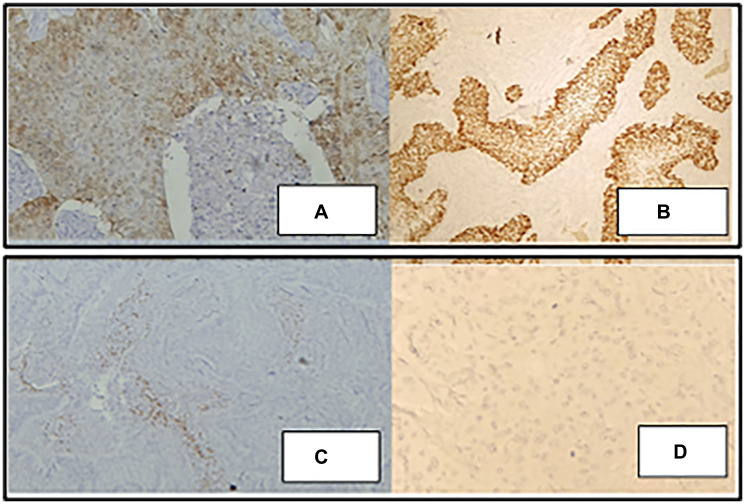
(**A**) shows p16 positivity, (**B**) shows p63 positivity, (**C**) shows ER negativity, (**D**) shows PR negativity.

## DISCUSSION

We present a rare case of superficial spreading SCC of the cervix involving the endomyometrium in a postmenopausal woman. The clinical presentation of superficial SCC is often nonspecific, with postmenopausal bleeding being a common symptom, as was seen in this patient [3]. Other reported symptoms in such cases include pyometra and vaginal discharge, both of which were absent in our case.

### Role of magnetic resonance imaging (MRI) in diagnosis

MRI is the preferred imaging modality due to its superior soft-tissue resolution. It plays a crucial role in the early detection and staging of cervical cancers, offering superior diagnostic accuracy compared to other imaging modalities [4]. MRI significantly outperforms clinical examination in estimating tumor size, especially in early-stage cervical cancers and cases with bulky cervical or endocervical lesions [5]. It aids in determining cancer stages and evaluating prognosis. Additionally, MRI is utilized to monitor treatment responses, track the effects of radiotherapy, and conduct follow-ups after treatment.

The staging of cervical cancer before treatment is essential for formulating an effective treatment strategy, with MRI serving a vital role in this assessment. The International Federation of Gynecology and Obstetrics (FIGO) staging system 2009 relied only on clinical evaluations. It did not consider lymph node involvement, resulting in inaccuracies in detecting advanced disease through imaging. The updated FIGO 2018 staging includes imaging results for the pre-treatment evaluation. Accurate local staging requires an assessment of several key elements: the depth of stromal invasion, the size of the tumor, whether there is invasion into the parametrium, vagina, or pelvic sidewall, any impact on the bladder or rectum, and the status of lymph node metastasis [6].

Tumor size measurements obtained through MRI may be affected by inflammatory edema or hemorrhage after biopsy procedures. The incorporation of advanced imaging modalities, such as diffusion-weighted imaging (DWI) and dynamic contrast-enhanced (DCE) sequences, has notably enhanced the diagnostic efficacy of conventional MRI [7]. These advancements contribute to increased accuracy in assessing tumor dimensions, lymph node involvement, parametrial invasion, and the detection of recurrence, thereby offering improved visualization of local tumor spread.

When supplemented with DWI and apparent diffusion coefficient (ADC) mapping, MRI provides a more obvious distinction between benign and malignant gynecological lesions. In the case of superficial SCC, DWI is beneficial, as it can differentiate tumor spread from normal surrounding tissues. The cancer typically shows high signal intensity on DWI and corresponding low signal intensity on ADC maps, indicative of a high cellular density.

MRI demonstrated the typical findings of superficial spread in our patient, with a hyperintense lesion on DWI in the endocervical region and corresponding low signal intensity on the ADC map. These imaging features suggested an extension of the cervical carcinoma into the uterine body, which was later confirmed by histopathological analysis. Importantly, the incorporation of MRI and DWI improved both diagnostic precision and tumor staging, enabling upstaging from stage IA to stage IB3 and thereby facilitating a more informed and appropriate treatment strategy for the patient. Such imaging findings underscore the utility of MRI and DWI as indispensable tools in the evaluation and management of early-stage cervical cancer, especially when there is suspicion of superficial spread to adjacent structures such as the endometrium and myometrium.

Comparable clinical and MRI findings have been described in a few published reports. Adler et al. observed atypical MRI features characterized primarily by focal endometrial thickening. Bagde et al. reported MRI findings of a fluid-filled endometrial cavity with irregular, circumferential thickening of the endometrium demonstrating intermediate-to-high signal intensity, surrounded by a thin hypointense rim involving the fundus, body, and lower uterine segment. Chitturi et al. described a pelvic MRI revealing an ill-defined T1 hypointense and T2 hyperintense lesion within the endocervical canal, extending superiorly to the lower third of the uterine body and infiltrating both anterior and posterior myometrial walls, resulting in the loss of the normal junctional zone. Similarly, Rangakar et al. reported an endocervical infiltrative, heterogeneously enhancing mass involving the cervix, associated with cervical stenosis and hydrometra.

Additionally, irregular thickening with nodular enhancing deposits, demonstrating restricted diffusion and contiguous extension from the cervical lesion along the endometrial lining, was also noted. An obvious cervical growth or lesion was not seen in all these studies. The MRI findings of reported cases of superficially spreading SCC of the cervix are illustrated in Table 1 [8].

**Table 1:** Published cases of MRI features of superficially spreading cervical SCC

Author (year)	Intramural extension or nodules	Irregular or thickened endometrium	Intrauterine or cervical fluid collection	Cervical tumor	DWI/ADC	Contrast-enhanced MRI	References
Adler et al. (2007)	−	+	+	+	NA	+	[9]
Bagde et al. (2021)	−	+	+	+	NA	NA	[10]
Narui et al. (2022)	Multiple	+	+	+	High/low	NA	[11]
Dokic et al. (2022)	−	−	−	+	NA	+	[12]
Shu et al. (2022)	−	−	+	−	NA	−	[13]
Mannan et al. (2022)	−	−	+	−	NA	NA	[14]
Takahashi et al. (2024)	Multiple	+	+	−	High/low	NA	[8]
Chitturi R et al. (2024)	+	+	−	+	NA	NA	[15]
Rangankar et al. (2024)	+	+	−	+	High/low	+	[16]
Patel et al. (2025)	−	−	huge hydrometra	−	NA	NA	[17]
Our study	+	+	+ (intracervical)	+	High/low	+	

Abbreviations: MRI: magnetic resonance imaging; SCC: squamous cell carcinoma; DWI: diffusion-weighted imaging; ADC: apparent diffusion coefficient; NA: not available.

### Pathological considerations

The typical patterns of uterine corpus involvement by cervical cancer are through deep myometrial penetration and lymphatic dissemination. Superficial spreading SCC is a different entity in terms of risk factors, clinical profile, and pathogenesis. Some risk factors or precursor lesions, like HPV infection, pyometra, endometritis, radiation, intrauterine devices, and exogenous or endogenous estrogens, have been postulated. The horizontal theory and vertical theory explain this unusual phenomenon. Cullen and Ferenczy et al. postulated the process of horizontal spread by which the neoplastic cells mechanically displace and eventually replaced the normal glandular epithelium of the endometrium [13, 18]. The replacement of normal healthy endometrium by superficial SCC can be evident on gross inspection as white patches. This hallmark finding is called “cake icing” or “Zuckerguss” carcinoma [18]. The second mechanism is a process of vertical proliferation (Field theory of carcinogenesis) by which the transformation of a normal cell into a malignant cell occurs in a vertical direction, with carcinoma in the cervix and the endometrium arising independently and concurrently by the same cancer-stimulating agent. The possibility of both mechanisms being operative is conceivable. In most of the reported cases, it is apparent that the endometrial lesion is a result of direct extension of the cervical cancer [17]. Histological continuity between cervical, endometrial, and fallopian tube lesions is often demonstrated, as seen in our case. Immunohistochemistry (IHC) analysis with four markers, p16, p63, ER, and PR, was done in our case. In line with our case, superficial spreading squamous cell carcinoma (SCC) of the cervix shows strong p16 immunoreactivity [19, 20], an established surrogate marker for high-risk human papillomavirus (HR-HPV) infection. HR-HPV plays a pivotal role in the pathogenesis of cervical SCC. Notably, one study [3] reported universal HPV16 positivity among all analyzed cases, reinforcing the association between persistent HR-HPV infection and the development of superficial spreading cervical SCC. p16 serves as a marker for HPV 16 infection in the uterine cervix; however, its sensitivity in predicting lesion progression is limited.

In contrast, Ki-67 and p63 are more informative in assessing whether a lesion is undergoing inflammatory metaplasia or atypical transformation. Elevated Ki-67 expression indicates neoplastic progression, while p63 can aid in distinguishing benign from malignant lesions. The combined use of p16 and p63 has demonstrated promising utility in predicting lesion progression [20].

Histopathological examination, in this case, was pivotal in differentiating between primary and secondary endometrial SCC. According to Fluhmann’s diagnostic criteria for distinguishing primary endometrial squamous neoplasia from secondary neoplasia, the diagnosis of primary endometrial SCC requires the absence of (a) coexisting endometrial adenocarcinoma, (b) a demonstrable connection between the endometrial tumor and stratified squamous epithelium of the cervix, and (c) primary cervical SCC [21]. In this case, the presence of contiguous spread from the cervix to the endometrium, as seen on MRI, and the histopathological findings supported a diagnosis of secondary endometrial involvement by cervical SCC. Immunohistochemistry further corroborated this diagnosis [14]. The tumor cells were positive for p16 and p63, markers indicative of HPV-related squamous cell carcinoma, while negative for ER and PR, which ruled out primary endometrial carcinoma. Immunohistochemistry in this context was essential to confirm the cervical origin of the tumor and exclude a primary endometrial neoplasm [15].

### Surgical management and prognosis

Jiang et al. reported 15 cases of superficially spreading squamous cell carcinoma (SCC) of the cervix, representing the most recent case study on superficially spreading cervical SCC published in PubMed [3]. Their study highlighted the clinicopathological features and treatment strategies in this rare entity. Most women were between 50 and 60 years of age (53.3%). Clinical presentation was heterogeneous, with abnormal vaginal bleeding being the most frequent symptom. Histopathological analysis revealed that most tumors were moderately differentiated SCCs, and all patients were positive for HPV16 infection.

The predominant histological subtype was cervical SCC with high-grade squamous intraepithelial lesion (HSIL), identified in 12/15 cases (80.0%). Additionally, endometrial HSIL with SCC was observed in 6 of 15 patients (40.0%). Treatment approaches varied: most underwent surgery followed by adjuvant therapy, including radiotherapy or chemotherapy (five patients received combined chemoradiotherapy and six received chemotherapy alone). Four patients, like ours, did not receive adjuvant treatment.

Comparable to our findings, cases of SCC with superficial extension into the endometrium, myometrium, and/or fallopian tube have been documented and are summarized in Table 2.

**Table 2:** Reported cases of superficial spreading SCC of cervix involving the endometrium and/or myometrium and/or salpinx and outcomes

Authors (year)	Age years	Clinical presentation	Cervical lesion	Extension into Endometrium, Myometrium, Fallopian tube	Adjuvant therapy	Outcome Follow-up (months)	References
Kushima et al. (2001) 2 Cases	78	Vaginal bleeding	Invasive	Endometrium (invasive), vagina (in situ)	NA	NA	[22]
	59	Vaginal bleeding, Lower abdominal mass	Invasive	Endometrium (in situ with endometrial stroma sarcoma), left fallopian tube, left ovary, vagina (in situ), vulva (in situ)	NA	NA	
Tan et al. (2004)	70	Vaginal discharge	Microinvasive	Endometrium (in situ)	−	NED (6)	[23]
Fadare et al. (2006)	38	Vaginal discharge	Invasive	Endometrium (in situ)	−	NED (9)	[24]
Adler et al. (2007)	59	Lower abdominal mass	Invasive	Endometrium (invasive)	NA	NA	[9]
Gungor et al. (2011)	53	Vaginal bleeding	Invasive	Endometrium (in situ, focal myometrial involvement), bilateral tubes and ovaries (in situ)	−	NED (12)	[25]
Marwah et al. (2012) 3 Cases	65	Pyometra	Invasive	Endometrium (in situ)	NA	NA	[26]
	60	Vaginal bleeding	Invasive	Endometrium (in situ with small focal microinvasion)	NA	NA	
	49	Vaginal bleeding	Invasive	Endometrium (in situ)	NA	NA	
Ishida et al. (2013) 2 Cases	64	Vaginal bleeding	Invasive	Endometrium (in situ)	NA	NA	[27]
	59	Vaginal bleeding	Invasive	Endometrium (in situ)	NA	NA	
Gulati et al. (2013)	50	Vaginal discharge	Invasive	Endometrium (invasive)	−	NED (6)	[28]
Sood et al. (2017)	60	Abdominal mass	Invasive	Endometrium (in situ)	NA	NA	[29]
Bagde et al. (2021)	63	Vaginal discharge	Invasive	Endometrium (invasive)	−	NED (6)	[10]
Martín-Vallejo et al. (2022)	66	Vaginal discharge	Invasive	Endometrium (in situ)	−	NED (12)	[30]
Dokic M et al. (2022)	62	Vaginal bleeding	Microinvasive	Endometrium (microinvasive) myometrium (microinvasive) right salpinx (microinvasive)	NA	NA	[12]
Narui et al. (2022)	50	Abdominal mass	Invasive	Endometrium (invasive) bilateral tubes (invasive)	NA	NA	[11]
Jiang et al. (2024) 4 Cases	71	Vaginal bleeding	Invasive	Endometrium (microinvasive)	−	NED (11)	[3]
	66	Vaginal discharge	Microinvasive	Endometrium (invasive)	−	NED (21)	
	81	Vaginal bleeding	Invasive	Endometrium (invasive)	−	NED (32)	
	76	Vaginal discharge	Invasive	Endometrium (invasive)	−	NED (49)	
Patel S et al. (2025)	80	Abdominal mass	Invasive	Endometrium, Myometrium >50%(invasive)	+ Chemoradiation	NED (6)	[17]
Nguyen et al. (2025)	45	Postcoital bleeding Pelvic pain	Invasive	Endometrium (invasive) myometrium (invasive) left salpinx (invasive)	+ Radiotherapy	NED (16)	[32]
Our Study	54	Vaginal bleeding Pelvic pain	Invasive	Endometrium (invasive) myometrium <50% (invasive) left salpinx (invasive)	−	NED (18)	

Abbreviations: NA: not available/reported; −: no adjuvant therapy; NED: no evidence of disease.

The patient underwent abdominal radical hysterectomy (ARH) with no adjuvant chemoradiation, given the favorable postoperative histopathological findings, including the absence of deep stromal invasion and lymphovascular space invasion [6]. The choice of ARH as a treatment modality for patients with FIGO 2018 stage IB3 cervical cancer has shown promising results, with studies indicating superior overall survival (OS) and disease-free survival (DFS) rates compared to patients treated with radiotherapy and chemoradiotherapy (R-CT)[31]. This outcome diverges from the recommendations outlined in the National Comprehensive Cancer Network (NCCN) guidelines, which often prioritize radiotherapy-based approaches for stage IB3 cervical cancer.

The current case exemplifies the potential oncologic advantages of ARH in selected patients with early-stage cervical cancer, particularly those with superficial spread. Nonetheless, this observation underscores the necessity for well-designed prospective clinical trials to determine the most appropriate management approach for patients with superficial spread of cervical carcinoma into the endomyometrium. Factors such as a prior history of cervical pathology, histopathological grade of the tumor, presence of lymphovascular invasion, and lymph node metastasis, in addition to tumor volume, play a crucial role in individualizing treatment strategies.

## CONCLUSIONS

This case report underscores the importance of thorough imaging and histopathological evaluation in managing cervical SCC with atypical spread patterns. Superficial spreading of SCC of the cervix to the endometrium is an uncommon phenomenon, but it carries significant implications for both staging and treatment. The use of MRI with DWI, coupled with comprehensive histopathological analysis, is crucial for accurate diagnosis and optimal treatment planning. In patients with FIGO 2018 stage IB3 cervical cancer, ARH appears to offer favorable oncologic outcomes, though further research is warranted to refine treatment guidelines for such cases. The role of immunohistochemistry in differentiating primary from secondary endometrial involvement remains indispensable in guiding clinical management.
